# A Centralized EHR-Based Model for the Recruitment of Rural and Lower Socioeconomic Participants in Pragmatic Trials: A Secondary Analysis of the Diuretic Comparison Project

**DOI:** 10.1001/jamanetworkopen.2023.32049

**Published:** 2023-09-01

**Authors:** Cynthia Hau, Jimmy T. Efird, Sarah M. Leatherman, Oleg V. Soloviev, Peter A. Glassman, Patricia A. Woods, Areef Ishani, William C. Cushman, Ryan E. Ferguson

**Affiliations:** 1 VA Cooperative Studies Program Coordinating Center, Boston, Massachusetts; 2 Department of Radiation Oncology, School of Medicine, Case Western Reserve University, Cleveland, Ohio; 3 Department of Biostatistics, Boston University School of Public Health, Boston, Massachusetts; 4 Pharmacy Benefits Management Services, Department of Veterans Affairs, Washington DC; 5 VA Greater Los Angeles Healthcare System, Los Angeles, California; 6 David Geffen School of Medicine at UCLA, Los Angeles, California; 7 Minneapolis VA Healthcare System, Minneapolis, Minnesota; 8 Department of Medicine, University of Minnesota, Minneapolis; 9 Medical Service, Memphis VA Medical Center, Memphis, Tennessee; 10 Department of Preventive Medicine, University of Tennessee Health Science Center, Memphis; 11 Department of Medicine, Boston University Chobanian and Avedisian School of Medicine, Boston, Massachusetts

## Abstract

**Importance:**

Participant diversity is important for reducing study bias and increasing generalizability of comparative effectiveness research.

**Objective:**

Demonstrate the operational efficiency of a centralized electronic health record (EHR)-based model for recruiting difficult-to-reach participants in a pragmatic trial.

**Design, Setting, and Participants:**

This comparative effectiveness study was a secondary analysis of Diuretic Comparison Project, a randomized clinical trial conducted between 2016 and 2022 (mean [SD] follow-up, 2.4 [1.4] years) comparing 2 commonly prescribed antihypertensives, which used an EHR-based recruitment model. Electronic study workflows, in tandem with routine clinical practice, were adapted by 72 Veteran Affairs (VA) primary care networks. Data were analyzed from August to December 2022.

**Main Outcomes and Measures:**

Measures reflecting recruitment capacity (monthly rate), operational efficiency (median time for completion of electronic procedures), and geographic reach (percentage of patients recruited from rural areas) were examined.

**Results:**

A total of 13 523 patients with hypertension (mean [SD] age, 72 [5.4] years; 13 092 male [96.8%]) were recruited from 537 outpatient clinics. Approximately 205 patients were randomized per month and a median of 35 days (Q1-Q3, 23-80 days) was needed to complete electronic recruitment. The annual income was below the national median for 69% of the cohort. Patients from all 50 states, Puerto Rico, and the District of Columbia were included and 45% resided in rural areas.

**Conclusions and Relevance:**

In this secondary analysis of a multicenter pragmatic trial, a centralized EHR-based recruitment model was associated with improved participation from underrepresented groups. These participants often are difficult to reach, with their exclusion potentially biasing trial results; eliminating in-person study visits and local site involvement can minimize barriers for the recruitment of patients from rural and lower socioeconomic areas.

**Trial Registration:**

The Diuretic Comparison Project (DCP) was registered on ClinicalTrials.gov Identifier: NCT02185417

## Key Points

**Question:**

Can a centralized electronic health record (EHR)-based model be used to successfully recruit participants from rural areas and with lower socioeconomic status, as evaluated by comparative effectiveness research?

**Findings:**

In this comparative effectiveness study and secondary analysis of a pragmatic randomized clinical trial, a geographically diverse cohort of 13 523 US patients with hypertension were efficiently recruited from all 50 states, Puerto Rico, and the District of Columbia. Approximately 45% of the cohort resided in rural areas and 69% had a lower socioeconomic status.

**Meaning:**

These results suggest that application of a centralized EHR-based model can reduce disparities in pragmatic clinical trials by increasing enrollment capacity, efficiency, and the inclusion of underrepresented groups.

## Teaser

This comparative effectiveness study and secondary analysis of a randomized clinical trial of US patients with hypertension examines the effectiveness of a centralized electronic health record–based model for successfully recruiting participants from rural areas and with lower socioeconomic status.

## Introduction

Pragmatic comparative effectiveness studies present an efficient alternative to randomized clinical trials (RCTs) when legacy electronic health records (EHR) are available.^1,2^ By using readily available real-world clinical evidence, this approach reduces the time and cost of conducting large-scale RCTs.^1,3,4^ Importantly, the properties of RCTs are preserved, including the minimization of study biases and the ability to make causal inferences independent of potential confounding.^5^ A pragmatic trial also helps to streamline patient recruitment, especially when implemented at the initiation of a study.^6,7,8^

Study recruitment can be embedded seamlessly into clinical workflows.^8^ This precludes the need for patients to attend in-person visits for clinical assessments. Alternatively, patients are recruited through routine health care.^9^ This is critical for a pragmatic study that aims to inform best treatment practices, as it promotes the inclusion of underrepresented groups that often are difficult to reach through conventional design.

We illustrate this concept in the context of the recently completed Diuretic Comparison Project (DCP).^10,11,12,13^ To our knowledge, this was the first nationally representative pragmatic trial to implement a clinically integrated recruitment design. Trial procedures were performed electronically and local site involvement was not required for recruitment. In this comparative effectiveness study, we discuss the operational details underlying the successful recruitment for DCP, specifically positing that an integrated recruitment design increases patient diversity and representation from rural and lower socioeconomic populations.

## Methods

### Electronic Recruitment Design

The Veterans Health Administration (VHA) is a health care system with approximately 1300 clinics across the US, delivering care to 9 million veterans yearly.^14^ To utilize the large amounts of patients and clinical data, trial procedures were embedded within the Veteran Affairs (VA) EHR systems. Electronic study procedures (omnibus approval for patient screening and customized patient-level randomization prompts) were embedded within the computerized patient record and physician order entry systems to operationalize recruitment workflow. Screening and recruitment data were extracted from the VA Corporate Data Warehouse (CDW), which is a centralized repository to combine and store EHR data generated from VHA clinics. This project was approved by the VA central institutional review board. The study operated under a waiver of written documentation of informed consent and a full waiver of Health Insurance Portability and Accountability Act authorization. However, verbal consent was obtained from eligible participants.^10,11^ The DCP followed the Consolidated Standards of Reporting Trials (CONSORT) reporting guidelines for RCTs, as reported in our previous publications.^10,11^

Trial recruitment for the DCP (conducted between 2016 and 2022; mean follow-up, 2.4 years) was performed in tandem with routine primary care.^10^ Following approval from primary care leaders at regional practices, the centralized recruitment system was applied to recruit their primary care clinicians (PCCs) and patients until 13 500 patients were randomized.

### Electronic Workflow

PCCs on active service were identified using the Primary Care Management Module (PCMM). This module captured records of clinical services provided by the VA primary care teams. Electronic consent from a particular PCC was collected through EHR. Once PCCs agreed to participate, a predefined algorithm was applied to assess patients’ eligibility for study participation (Box). A recruitment letter was sent to eligible patients, as well as instructions for submitting a voicemail opt-out request to decline study participation. If the patient did not refuse to participate in the study (by voicemail message), their telephone information was included in the contact list and forwarded to the central calling centers. Each attempt to contact the patient by telephone and verbal consent were documented in the study database, allowing research staff to monitor patient enrollment status.

Box. **EHR-Based Eligibility Definitions**

**Eligibility Criteria**

1. Age ≥65 y
2. Receiving hydrochlorothiazide at 25 or 50 mg daily (within 25% from the targeted values)
3. Systolic blood pressure ≥120 mm Hg (most recent record within 2 y of data extraction date)
4. In the past 90 d, no record of systolic blood pressure <120 mm Hg
5. In the past 90 d, no record of potassium <3.1 or <3.5 mEq/L (if receiving digoxin medication)
6. In the past 90 d, no record of sodium level <130 mEq/L

To convert potassium to millimoles per liter, multiply by 1.0; sodium to millimoles per liter, multiply by 1.0.

PCC approval was required before patient randomization. An electronic order was sent to request a PCC’s assent for a particular patient to undergo study randomization. The PCC provided an electronic signature (using the physician order entry feature) to confirm approval. The corresponding electronic approval was detected from a patient’s EHR. Medical record review was performed by study nurses to verify patient eligibility. Once confirmed, electronic randomization was performed.

For patients who were randomized to continue with hydrochlorothiazide, no further action was needed from their PCC. However, de facto PCC approvals were needed if the patients were randomized to switch from hydrochlorothiazide to chlorthalidone (ie, changing medication from usual care practice). An EHR alert notification was sent by the study nurse, requesting the PCC to discontinue hydrochlorothiazide and prescribe chlorthalidone. PCC approval was provided as an electronic signature, which was detected directly from CDW. The release of chlorthalidone medication was subsequently confirmed by EHR data generated from the VA outpatient pharmacy services. Notification letters were sent to patients by the central mailing center when a PCC declined study randomization, study nurses determined patients were ineligible based on EHR review, or a PCC approved study randomization and signed the corresponding prescription orders.

A web-based software application (ProjectFlow) was applied to support custom research data collection,^15^ including insertion of timestamps to mark process completion. Collected data were transferred to a structured query language (SQL) server where the study database was stored. In addition, the data collection application was used to display recruitment progress on a web-based interface, allowing callers and study nurses to monitor patient status with point-and-click queries. A data integration technique, the extract-transform-load (ETL), was used to process raw data (EHR and ProjectFlow) and uploaded to the study database.

**Figure 1.**
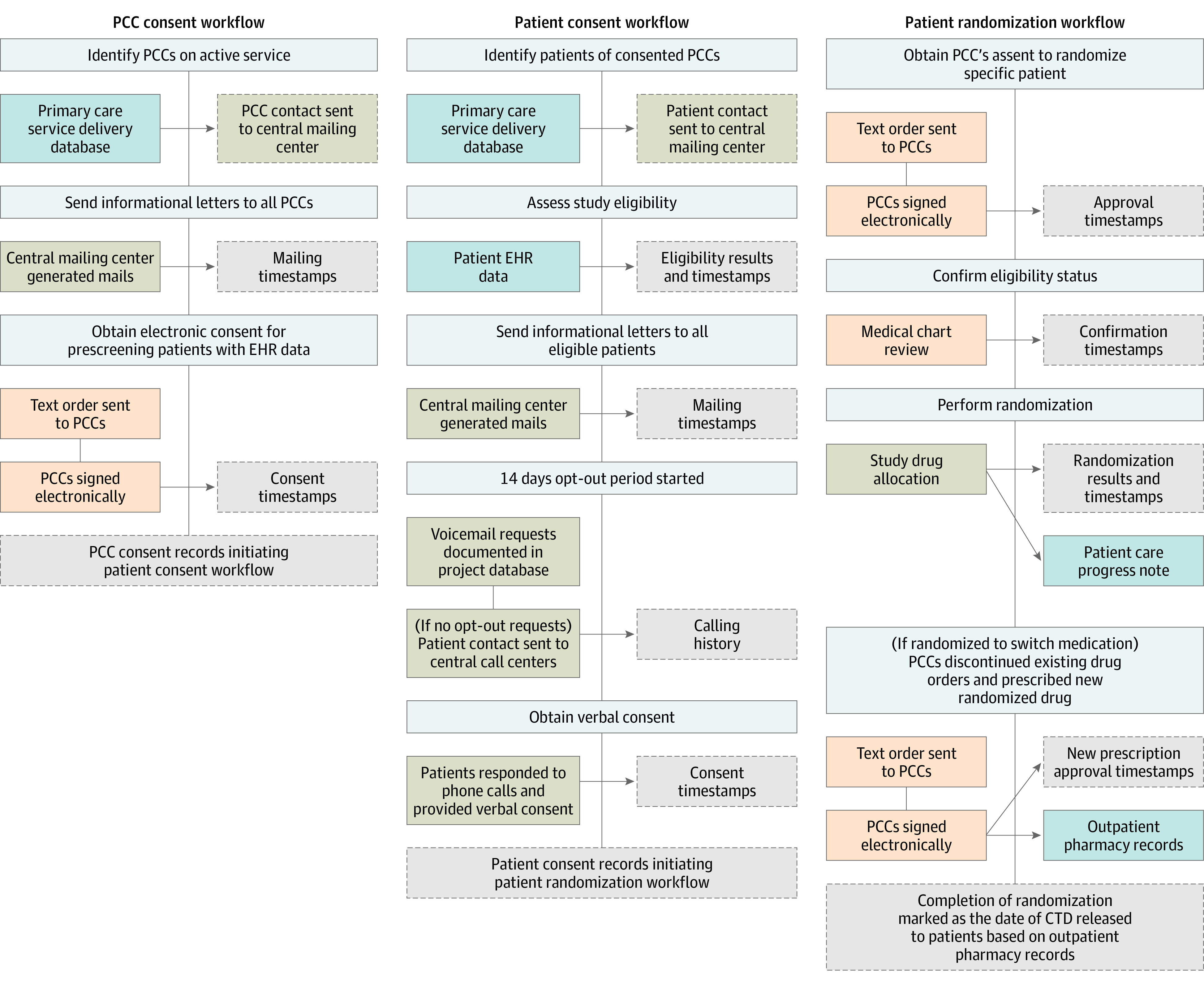
Centralized Electronic Health Record (EHR)-Based Recruitment Workflow for the Diuretic Comparison Project (DCP) The architectural features that distinguished our model from other systems were its adaptability and stability, as partitioned into 3 electronic workflows (primary care clinician [PCC] consent, patient consent, and patient randomization). This is accordingly delineated in the top-down, tree-like flow of this diagram. Blue boxes indicate a process embedded in the VA Cooperative Data Warehouse (CDW); beige, research data captured in the web-based software used to customize data collection; orange, tasks managed by study nurses through computerized patient record system; dark gray, creation of study database; and light gray, standard trial procedures to enroll PCCs and patients.

Electronic operations were standardized across the VA primary care networks (Figure 1). First, for PCC consent workflow, contact information was extracted among PCCs whose employment status was labeled with an active flag. Mailing timestamps were generated by data collection application after an informational letter was sent. An electronic consent process was triggered after 7 days from the mailing date. Response to the consent request was captured with a list of entry options (ie, lapse, accept, or decline). Consented PCCs were defined as those who accepted the order for prescreening their patients with EHR data, initiating the consent workflow.

Patients managed by the consented PCCs were then identified and assessed for study eligibility. Contact information of eligible patients were loaded into the web application. A mailing timestamp was generated after an informational letter was sent, which marked the 14 days opt-out period. If an opt-out timestamp was absent, each patient’s telephone information was loaded into the application for the central calling centers. Call attempts and consent status were documented. Corresponding consent records were transferred to the SQL study database, which triggered the randomization workflow among consented patients.

Study randomization approval obtained from PCC were documented in patient’s EHR. A daily tracking of the specific EHR data (PCC’s electronic approval) was automated by applying a real-time signature alert. A predefined order entry was used when requesting PCC approval. The digital approval was detected with a string-matching algorithm. This technique also was used to search PCC approval for hydrochlorothiazide discontinuation and chlorthalidone prescription.

### Validations

A standardized validation process was performed at the regional level, which involved a clinical applications coordinator (CAC) from each location. The CAC is a technician who supports local software integration. During the validation process, the CAC was asked to create a small set of test PCCs and patients (simulating real data presented in EHR). All algorithms and ETL processes were tested by the research staff. Testing included identifying test PCCs and patients from EHR data, documenting eligibility and consent based on assigned status, and detecting approval orders when digital signatures were provided. These steps were necessary to ensure the electronic workflows were compatible with regional practices.

The Boston Healthcare System was the first primary care network to implement these electronic workflows. Changes were anticipated with the inclusion of other regional networks. Accordingly, the EHR integrations were assembled using a modular design to minimize programming effort during system modification. Database updates also were expected. This entailed data format changes and other modifications to the data structure. The research staff had limited control over these changes that were implemented by the EHR system developers. A series of quality control measures were needed to identify unpredictable changes.

### Statistical Analysis

The DCP recruitment results were reviewed to discuss the potential benefits with centralized operations. Measures reflecting recruitment efficiency (time to randomization and consent) were examined. Overall duration of completing each electronic procedure was reported as medians and Q1-Q3 ranges. Means (with SDs) were reported for the monthly rate of electronic consent and randomization.

**Figure 2.**
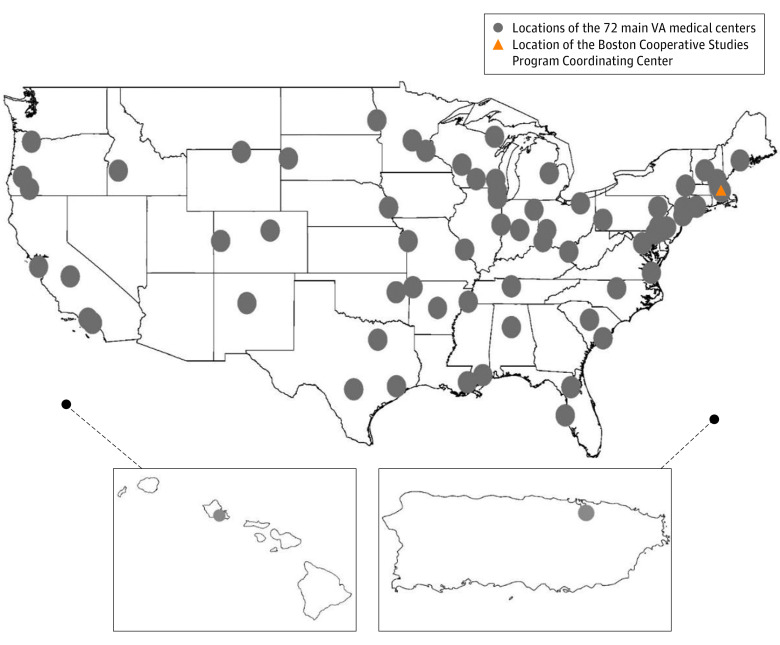
Geographic Reach of the Diuretics Comparison Project (DCP) Locations of electronic recruitment were graphically displayed, with the triangle indicating the location of a centralized study coordinating center where the monitoring of trial recruitment occurred, and dots the main VA medical centers responsible for acquiring patient data from the regional VA primary care networks. Each of the centers act as the repository of data generated within its respective region, including all affiliated primary care clinics.

**Figure 3.**
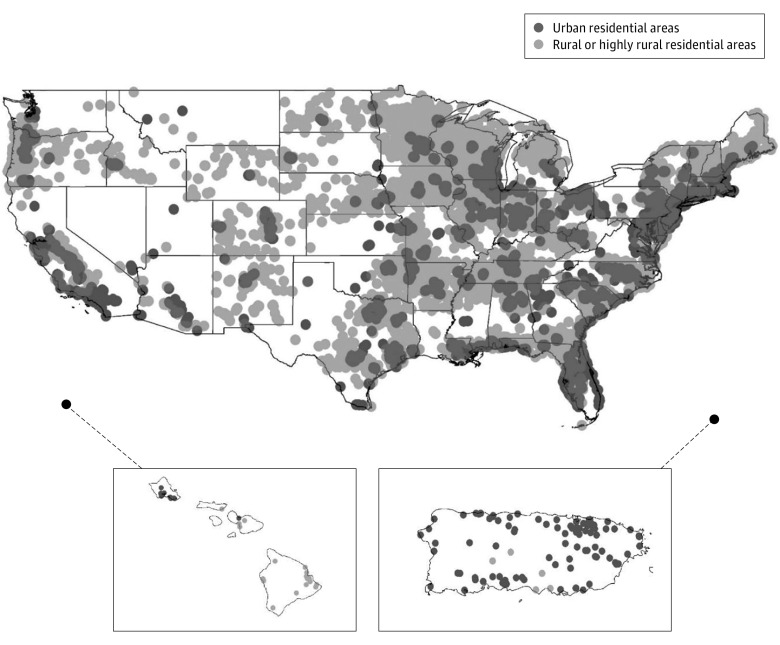
Geographic Distribution of Randomized Patients Across the US Residential locations of 13 523 included patients were graphically rendered using the geographic information system data extracted from the VA Cooperative Data Warehouse (CDW). The CDW is a centralized repository to combine and store EHR data generated from all VA health care facilities. Dots correspond to participants residing in urban (dark shading) vs rural and highly rural (light shading) locations, but respectively do not infer density.

Recruitment locations have been graphically rendered using the geographic information system (GIS) software ArcMap version 10.5 (ESRI) (Figure 2). Patient residential locations were presented using the GIS data extracted from CDW, with VA classification of urban and rural areas (Figure 3).^16^ The median nonfamily household income was defined as $40 464 according to the 2020 US Census Bureau results.^17^ Numbers were reported to 2 significant digits, vs a fixed number of decimal places, using the Goldilocks (Efron–Whittemore) rounding method.^18^

## Results

### Recruitment Capacity

A total of 1 404 771 patients were eligible for electronic screening—14 743 were determined as eligible by study nurses and provided verbal informed consent with 13 523 (91.7%) consented completing randomization (mean [SD] age, 72 [5.4] years; 13 092 male [96.8%]). Of the 4128 consented PCCs, 2944 (71.3%) agreed to the randomization of at least 1 patient.

Patients across the US (including Puerto Rico and District of Columbia) were enrolled, demonstrating excellent geographic reach (Figure 3). Of the 13 523 patients randomized, 6126 (45.3%) resided in rural or highly rural locations, which compared favorably with traditional recruitment methods.^19^ Self-reported income was available for 8714 randomized patients, with 6013 (69.0%) having an annual income below the national median level for nonfamily households.

Although centralized monitoring and research staff were based in Boston, participants of each region of the US were well represented. Among those residing in urban areas (7371 participants), the highest percentages were observed in the South (2892 [39%]), followed by the Midwest (2224 [30%]), West (1114 [15%]), and Northeast (954 [13%]) regions, with the remaining 187 (3%) from Puerto Rico and the District of Columbia. Those residing in rural or highly rural areas (6126 participants) were primarily from the South (2338 [38%]), Midwest (2340 [38%]), West (809 [13%]), and Northeast (631 [10%]) regions, as well as Puerto Rico and the District of Columbia (8 [0.1%]).

### Recruitment Efficiency

**Figure 4.**
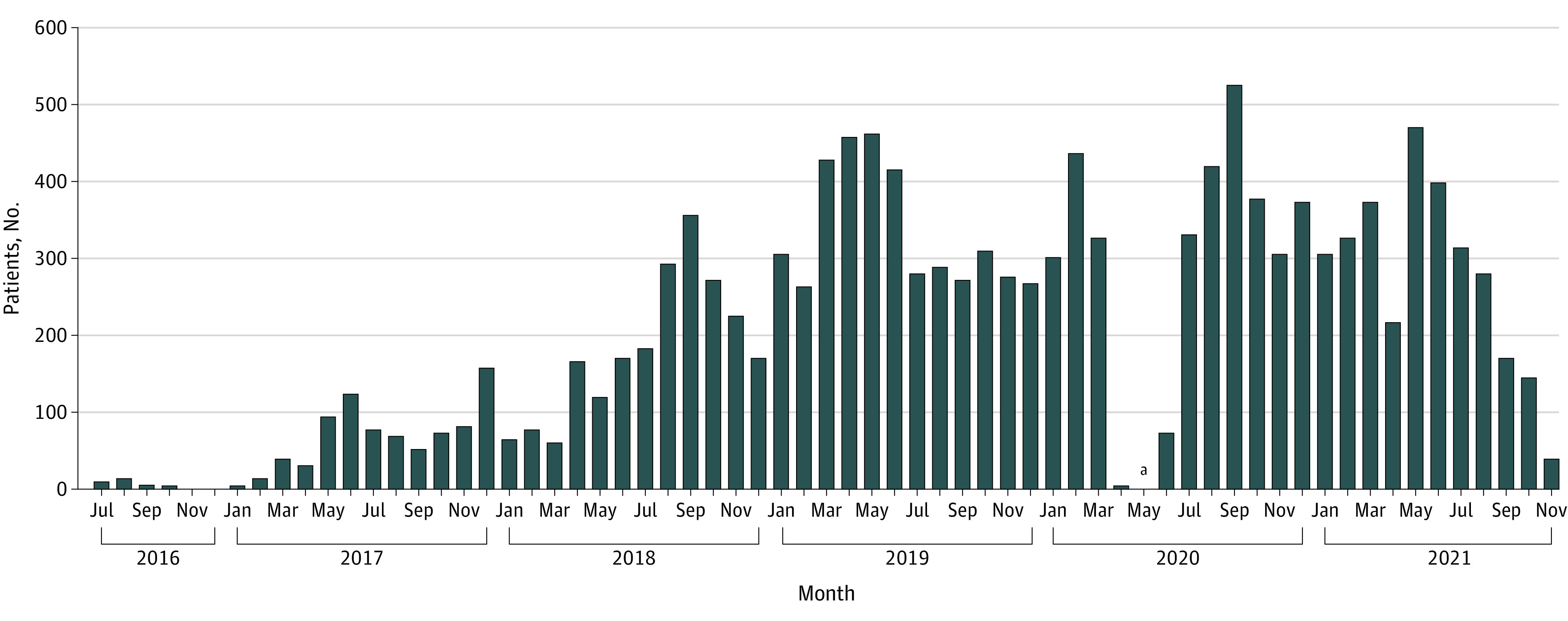
Recruitment of Patients for the Diuretic Comparison Project (DCP) Measure reflecting the electronic recruitment capacity (monthly rates) presented. An exponential increase from 2016 to 2021 was observed for the number of Veterans Administration primary care networks participating in the pragmatic DCP. ^a^Recruitment activity temporarily suspended due to the COVID-19 pandemic.

Recruitment occurred over a 5.4-year period (Figure 4). On average, a median (Q1-Q3) 14 (10-23) days were needed to consent PCCs. The patient consent and randomization processes were completed over a median (Q1-Q3) 35 (23-80) days, with a mean (SD) monthly enrollment rate of 205 (153) patients. The median (Q1-Q3) period from when patients were deemed eligible until consent records appeared in study database was approximately 24 (17-63) days. Next, text orders were sent to PCCs, with median (Q1-Q3) randomization approvals obtained over the following 5 (3-11) days. An additional median (Q1-Q3) 1 (0-2) day was required for PCCs to sign the electronic prescription orders if the patients were randomized to the chlorthalidone group.

From 2016 to 2021, an exponential increase was observed for the number of VA primary care networks participating in DCP (ranging from 1 to 72). An additional 18 networks joined in 2018 and 29 in 2019. The peak randomization rate was observed in 2019, with 4020 patients randomized that year.

## Discussion

A key emphasis of the current article is on recruitment and how a pragmatic design can positively affect the conduct and reach of a clinical trial. The centralized EHR-based recruitment model developed for DCP specifically enhanced recruitment capacity and efficiency, expanding the coverage of underrepresented patients (especially those living in remote and rural areas). In many cases, such participants may not have adequate financial, transportation, or mobility resources. Insufficient support for sensory impairments also may be challenging. The success of enrolling a diverse study population in DCP highlights the generalizability of study results and serves as a model for future research to inform evidence-based clinical practice.

Pragmatic trials typically are embedded within routine clinical practice. They can take full advantage of the technical advances that are widely available for increasing outreach to rural populations.^20^ This includes using centralized EHR to aid with clinical trial recruitment, ultimately translating into greater patient representation, improved study efficiency, shorter completion time, and reduced costs.

Utilizing EHR technology to complete trial requirements helps to offset tasks traditionally performed during in-person visits.^9,21^ Common applications include applying EHR integrations to assess patient eligibility, engage PCCs, and randomize targeted patients.^8,22,23^ Emphasizing the holistic application of EHR systems is important to streamline the entire recruitment process vs a fragmented approach. The latter is inefficient as it requires local site involvement to complete study recruitment. On average, the study randomized 187 patients from each of the 72 primary care networks, highlighted the capacity of the system to recruit from both small and large sized clinics (ranging from approximately 50 to 3000 patients).

### Management

Performing extensive review of EHR data for each patient was impractical. The process was labor-intensive and defeated the intent of a pragmatic trial. Therefore, data-driven reviews were undertaken to ensure data quality, conducting either through systematic adjudication of crosswalk tables or using a site-specific random-sampling approach.

The adjudication of the hydrochlorothiazide drug list was an example of a systematic review. Updates to the medication lists were performed yearly by the VA EHR vendor. These were electronic listings captured by CDW to document prescriptions supplied at the VHA facilities. Based on the frequency of update, annual review was performed by study nurses, to include new variations of drug names or doses to the extraction algorithm.

A random-sampling review approach was used to verify PCC and patient contact. This was done before applying the electronic workflows to a specific VA primary care network. Project nurses performed medical record review on a small sample, to verify contact information against data shown in CDW. This was necessary because information might be formatted differently across regional systems, yielding invalid recipient names or addresses pulled by the automated process. This preserved time from sorting returned mails and confirming that patients received appropriate notification letters.

Results originating from EHR data might be structured differently across primary care networks, even though the PCC orders were predefined and entered consistently. This resulted in the need to manually track PCC discontinuation and approval orders. For example, the organization of PCC orders might vary slightly between networks. When this occurred, the ETL process was unable to search for strings that contained the predefined text.

### Benefits

The configurations and templates developed for DCP are designed to be readily adaptable by future pragmatic studies. Most projects that utilized an EHR system for clinical research focused on applying a standalone system or software, which often are designed for singular trial use.^8^ However, with our approach, the pragmatic strategies learned from previous trials are carried over to the next study. This accelerates implementation of research within a health care system and contributes to the creation of a shared learning environment in clinical practice.

Using centralized recruitment was effective for enrolling patients from rural areas and lower socioeconomic backgrounds. Remote patients who had access to a primary care network were enrolled. An illustration of this was the State of Hawaii, with the main hospital located in Oahu (Figure 3). However, with health care network interconnected through the VA system, we were able to include patients from other Hawaiian Islands.

Another important benefit of centralized recruitment is reducing trial completion time while increasing the ability to compare drugs currently being prescribed by local PCCs. The trial reflects the most updated treatment options. In DCP, recruitment lasted 5.4 years, which was 2.4 years longer than originally projected. While there were unexpected delays from system setup and obtaining approvals from regional primary care leaders, the overall increased number of participating networks had a substantial effect on recruitment efficiency. By 2018, the DCP was able to randomize over 2000 patients yearly and recruitment rates were as high as 400 patients for certain months.

### Limitations

This study had several limitations. In addition to the typical challenges with secondary analysis of EHR data (eg, missing data or claims data being incompatible across systems), there were other unanticipated issues. A few patients were inappropriately randomized notwithstanding vigorous monitoring. In one case, a patient died shortly after providing informed consent. While the study nurse confirmed participant eligibility status prior to randomization, the death record was not yet available in the VA EHR system. Because in-person contact was not required with the centralized recruiting model, neither the PCC nor study team were aware of the death. Additionally, a few participants were subsequently found to be enrolled in more than one clinical trial. This was difficult to detect because the EHR system was not intended for clinical trial use.

The implementation and maintenance of an EHR-based workflow involves a multidisciplinary research team (eg, clinicians, medical informaticians, epidemiologists, biostatisticians, and data scientists). On some occasions, the study team may encounter EHR data problems that are difficult to trace. Although more resources are spent on technological support, it is still worthwhile to apply a centralized EHR-based recruitment model when conducting a pragmatic trial.

## Conclusions

Increasing recruitment from underrepresented groups is an important problem to address for RCTs. This was innovatively accomplished without the need for local site involvement. The pragmatic approach is a relatively new concept in the conduct of RCTs. Importantly, our step-by-step approach and detailed workflows provides an easy-to-follow blueprint for others seeking to implement point of care studies in the context of usual clinical care.

While an EHR-based pragmatic approach has important advantages over a traditional study design for patient recruitment, successful implementation and conduct are contingent on the technical expertise of team members and ongoing support from information services. However, we believe that the potential challenges encountered by health care systems to implement a pragmatic approach in the short run will be off set as newer technologies arise (eg, artificial intelligence, machine learning, and natural language processing).

## Notes

Supplement.
Data Sharing Statement
