# Supplementary material for: A Centralized EHR-Based Model for the Recruitment of Rural and Lower Socioeconomic Participants in Pragmatic Trials: A Secondary Analysis of the Diuretic Comparison Project
Source: JAMA Netw Open. 2023 Sep 1;6(9):e2332049. doi: 10.1001/jamanetworkopen.2023.32049 (PMC10474559; doi:10.1001/jamanetworkopen.2023.32049)
Supplement: Supplement. — Data Sharing Statement [file jamanetwopen-e2332049-s001.pdf]

Data Sharing Statement

Hau. A Centralized EHR-Based Model for the Recruitment of Rural and Lower Socioeconomic Participants in Pragmatic Trials. *JAMA Netw Open*. Published September 01, 2023. doi:10.1001/jamanetworkopen.2023.32049

Data
Data available: No

Additional Information
Explanation for why data not available: The datasets used or generated from the current study are not publicly available. De-identified, aggregated data may be provided upon request through an approved VA Data Use Agreement.
